# Congenital syphilis and oral involvement: *Treponema pallidum* detection and oral development in exposed children

**DOI:** 10.31744/einstein_journal/2026AO2472

**Published:** 2026-08-14

**Authors:** Lourenia Caroline Fernandes Veiga, Renata Cicci Cunha Castro, Nathan Castro Silva, Lucas Batista Tiago, Mateus Rodrigues Correa, Camilla Beatriz da Silva Melo, Caroline Ribeiro Castro Sousa, Vinicius Rangel Geraldo-Martins, Maria Angélica Hueb de Menezes, Ruchele Dias Nogueira

**Affiliations:** 1 Universidade de Uberaba Uberaba MG Brazil Universidade de Uberaba, Uberaba, MG, Brazil.

**Keywords:** Syphilis, congenital, Treponema pallidum, Oral manifestations, Polymerase chain reaction, Infant, newborn

## Abstract

**Objective:**

To investigate oral manifestations and the presence of *T. pallidum* DNA in children exposed to maternal syphilis by integrating epidemiological, clinical, and molecular data.

**Methods:**

This two-phase observational study analyzed health, perinatal, and dental records of children born to mothers diagnosed with syphilis during pregnancy. In Phase 1, demographic characteristics, maternal treatment adequacy, birth conditions, and oral clinical findings were assessed. In Phase 2, a subset of children underwent comprehensive oral examinations, including evaluation for enamel defects associated with congenital syphilis. Oral samples were collected and processed by DNA extraction followed by nested PCR targeting *T. pallidum*-specific genes.

**Results:**

Although no statistically significant differences were observed between maternal treatment groups for some outcomes, children exposed to maternal syphilis showed enamel hypoplasia, Hutchinson-like incisors, and molar defects. PCR amplification identified *T. pallidum* DNA in oral samples from a subset of participants, suggesting possible persistence of treponemal DNA or residual genetic material after maternal treatment.

**Conclusion:**

Children exposed to maternal syphilis may present with distinctive oral alterations, and molecular detection of *T. pallidum* DNA in the oral cavity supports the need for long-term dental follow-up and integration of oral examinations into postnatal care programs.

## Highlights

■Oral swabs were collected at birth and analyzed by nested polymerase chain reaction for *T. pallidum* DNA.

■A high prevalence of enamel hypoplasia, Hutchinson-like incisors, and molar defects was observed at 6-10 years of age.

■Molecular detection in oral samples suggests possible persistence of treponemal DNA or residual genetic material.

■Integrating oral examinations into postnatal care programs may support long-term monitoring of children exposed to maternal syphilis.

## In Brief

Children exposed to maternal syphilis were evaluated in two phases. At birth, oral swabs were collected and analyzed by nested polymerase chain reaction to detect Treponema pallidum DNA. At 6-10 years of age, clinical oral examinations revealed a high prevalence of enamel hypoplasia, Hutchinson-like incisors, and molar defects. Molecular detection of *T. pallidum* DNA in the oral cavity suggests possible persistence of treponemal DNA or residual genetic material, supporting the need for long-term dental follow-up and integration of oral examinations into postnatal care programs.

## Graphical abstract

**Figure:**
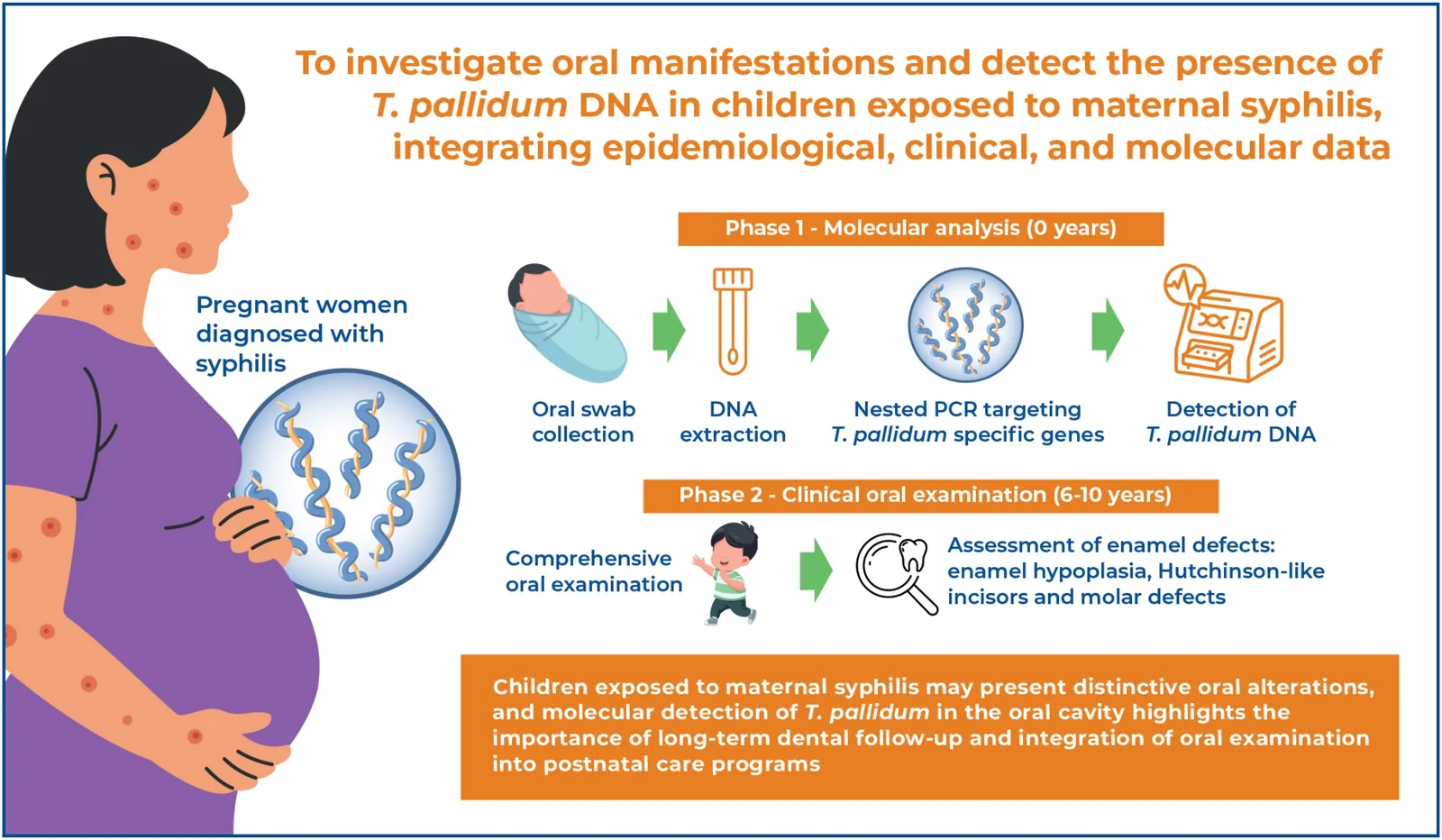

## INTRODUCTION

The global resurgence of syphilis, caused by the bacterium *Treponema pallidum* subspecies *pallidum*, represents an escalating public health concern.^(1,2)^ Although syphilis had been largely controlled in many regions, rates of primary, secondary, and particularly congenital syphilis (CS) have increased worldwide over the past decade, underscoring the need to re-evaluate diagnostic and surveillance strategies.^(3-5)^ Congenital syphilis results from transplacental transmission of the spirochete from an infected pregnant woman to the fetus. This transmission can lead to severe multisystem morbidity, long-term neurodevelopmental sequelae, stillbirth, or neonatal death.^(6,7)^ Importantly, CS is largely preventable through universal screening during pregnancy and timely penicillin treatment.^(8,9)^ The persistence of CS reflects gaps in antenatal care coverage, adherence to screening protocols, and timely treatment, especially in high-risk populations.^(10-12)^

The clinical presentation of CS is highly variable, which has led to its historical designation as "the great imitator."^(13)^ The severity and type of manifestations depend on the timing of maternal infection and treatment, the gestational stage at fetal infection, and the infant's age at presentation.^(14)^ Early manifestations, which typically occur within the first 2 years of life, include mucocutaneous lesions, such as syphilitic rhinitis and maculopapular rash, hepatosplenomegaly, osteochondritis, and hematological abnormalities.^(4)^ If untreated or inadequately treated, CS may progress to late manifestations after 2 years of age, affecting the central nervous system, eyes, bones, and craniofacial structures.^(15)^

Diagnosis is further complicated by this varied clinical presentation. Infected neonates may be asymptomatic at birth but develop complications later in life. Furthermore, conventional diagnosis relies largely on serological testing, including non-treponemal and treponemal tests, which may be difficult to interpret in newborns because of passively acquired maternal antibodies.^(2)^ This diagnostic ambiguity supports the exploration of complementary, non-invasive, and specific detection methods, particularly for evaluating possible latent infection or persistent microbial genetic material.

Oral and maxillofacial involvement is a recognized feature of late CS and can provide durable evidence of past infection.^(16,17)^ Dental stigmata are particularly relevant because treponemal infection may disrupt amelogenesis and odontogenesis during critical periods of tooth development. These classic defects include Hutchinson's incisors, defined as permanent maxillary central incisors that are characteristically peg-shaped and show a central crescent-shaped notch,^(18,19)^ and mulberry molars (Moon's molars), a malformation of the first permanent molars in which the occlusal surface appears lobulated because of cusp hypoplasia.^(20)^

These dental anomalies, together with other oral manifestations such as gummas and mucous patches, highlight the role of dental practitioners in the syphilis care continuum.^(21,22)^ Careful clinical evaluation is required to distinguish syphilitic dental defects from other developmental anomalies, including those associated with trauma, nutritional deficiencies, or historical mercury-based treatment.^(16,23,24)^ Continued reporting of oral manifestations, ranging from classic signs to rare isolated lesions, supports the value of the oral cavity as a diagnostic site for both acquired and congenital forms of syphilis.^(25-27)^

Although the clinical and pathological oral consequences of CS have been documented, limited molecular evidence is available regarding whether *T. pallidum* DNA can be detected in the oral cavity of exposed children.^(28,29)^ Syphilis primarily involves mucosal surfaces, and the oral mucosa is a common site of infectious lesions in primary and secondary acquired syphilis and early CS.^(29)^ The complex oral environment, including mucosal folds, salivary glands, dental pulp, and periapical tissues, may therefore represent a potential site for residual treponemal DNA detection, even after antibiotic treatment or in the absence of active serological markers.

Molecular detection methods, particularly polymerase chain reaction (PCR)-based assays targeting *T. pallidum* DNA, may help address this gap. Previous research has focused largely on epidemiological data and serology.^(12,30)^ Accordingly, this study focuses on molecular detection in the oral cavity, where classic CS-related dental stigmata may be observed.

## OBJECTIVE

To investigate oral manifestations and the presence of Treponema pallidum DNA in the oral cavity of children exposed to maternal syphilis by integrating clinical, epidemiological, and molecular findings to characterize the long-term consequences of congenital exposure.

## METHODS

### Study design and setting

The study protocol was approved by the Institutional Research Ethics Committee of the *Universidade de Uberaba* (CAAE: 36067220.0.0000.5145; # 4.717.090) and was conducted in accordance with the Declaration of Helsinki and Brazilian National Health Council Resolution No. 466/12. Written informed consent was obtained from the legal guardians of all participating children for sample collection and the confidential use of clinical data.

This two-phase observational study investigated the association between maternal syphilis during pregnancy and oral and systemic health outcomes in offspring. The study was conducted at the *Universidade de Uberaba* (UNIUBE).

Phase 1, the neonatal cohort, included 29 newborns aged ≤72 hours born to mothers with confirmed syphilis during pregnancy; 17 were born to mothers who completed treatment with benzathine penicillin, and 12 were born to mothers who were untreated during pregnancy. The control group included 10 newborns born to mothers without a syphilis diagnosis.

Phase 2, the childhood follow-up cohort, included children aged 6-10 years who were born to mothers from the same maternal cohort or were identified through medical records as having been exposed to maternal syphilis during gestation.

The inclusion criteria for Phase 1 were neonates born to mothers with a documented diagnosis of syphilis during the index pregnancy, confirmed by both non-treponemal and treponemal tests; age ≤72 hours at examination and saliva collection; and written informed consent from the mother or legal guardian. The inclusion criteria for Phase 2 were children aged 6-10 years born to mothers with documented syphilis during pregnancy and identified through hospital, public health, or laboratory databases; availability of parent/guardian contact information and willingness to participate; absence of systemic conditions contraindicating oral examination or saliva collection; and parental/guardian informed consent and child assent.

The exclusion criteria for both phases were systemic diseases unrelated to congenital syphilis that could alter oral conditions, such as genetic syndromes or chemotherapy exposure, and refusal or withdrawal of consent.

A structured questionnaire was administered to collect sociodemographic data, including maternal age, education, and socioeconomic status; prenatal history, including the number of antenatal visits, gestational age at diagnosis, syphilis serology results, venereal disease research laboratory/rapid plasma reagin titers, treponemal test results, and timing and type of treatment; substance use, including alcohol, tobacco, and drug use; and comorbidities.

Both phases involved clinical oral examinations and analysis of maternal, perinatal, and health data collected through structured questionnaires and medical records. Saliva was collected in Phase 1 for molecular detection of *Treponema pallidum* DNA.

### Phase 1 — Neonatal cohort procedures

#### Oral clinical examination

Neonates were examined by trained pediatric dental clinicians within 72 hours after birth using sterile gloves, headlamp illumination, and disposable tongue depressors. The examination assessed external and intraoral anatomy, including the lips, tongue, gingiva, palate, and mucosa; congenital oral lesions, including ulcers, papules, and mucous patches; natal or neonatal teeth; enamel defects or discoloration; and gingival enlargement or hemorrhagic changes. Findings were recorded on standardized forms, and photographs were obtained with parental permission.

### Saliva collection and molecular analysis

Saliva samples were collected by suction using graduated sterile pipettes, transferred to tubes, stored on ice, and processed in the Biopathology Laboratory. DNA was extracted from saliva samples using the PowerLyzer PowerSoil DNA Isolation Kit (MO-BIO, Carlsbad, CA, USA) according to the manufacturer's protocol. Samples were transferred to tubes containing Bead Solution, vortexed for 2 min to promote bacterial disruption, and subsequently transferred to PowerLyzer Glass Bead Tubes for mechanical lysis. DNA concentration and purity were determined using a NanoDrop 2000 spectrophotometer (Thermo Scientific).

Primers targeting T. pallidum DNA (Invitrogen, Carlsbad, CA, USA) were used for amplification: forward, 5′-GCG TGT TTT ATC GGT TGC TT-3′; reverse, 5′-CCA AAT AAA CTT CCT GTC TCC A-3′. Real-time polymerase chain reaction (PCR) assays were performed using a StepOne™ System (Thermo Fisher Scientific) with 6.5µL SYBR Green Master Mix (Roche, Illinois, USA), 1µL of each primer, 4.5µL ultrapure water, and 2µL extracted DNA. Cycling conditions consisted of initial denaturation at 95°C for 10 min, followed by 40 cycles at 95°C for 15 s and 60°C for 1 min. DNA from the T. pallidum Nichols reference strain (10^9^ CFU/mL) served as the positive control, sterile ultrapure water served as the negative control, and all reactions were performed in duplicate.

### Phase 2 — Follow-up cohort of children aged 6-10 years

#### Recruitment and follow-up

Children aged 6-10 years who were born to mothers with documented syphilis during pregnancy, with or without adequate treatment, were identified from neonatal records of Phase 1 participants and from congenital syphilis registries at Mário Palmério Hospital Universitário. Parents or guardians were contacted and invited to participate in the follow-up study at the Getúlio Vargas Polyclinic of UNIUBE. After informed consent and child assent were obtained, participants underwent clinical and questionnaire-based evaluations.

#### Questionnaire and general health assessment

Parents or guardians completed a structured questionnaire assessing the child's medical history, growth, and development; systemic conditions possibly related to congenital infection, including neurological, visual, or auditory deficits; history of antibiotic treatment; oral symptoms, including ulcers, delayed eruption, and dental pain; dietary habits; and oral hygiene practices. Pediatric medical records were reviewed, when available, to confirm systemic findings.

### Clinical oral examination of children

A comprehensive intraoral examination was performed by calibrated dental clinicians (Cohen's κ≥0.80). The protocol included dental evaluation for enamel hypoplasia, Hutchinson's i,ncisors, and mulberry molars; caries assessment using the decayed, missing, and filled teeth (DMFT) index; soft-tissue examination for gingival inflammation, hyperplasia, ulcerative or mucous lesions, and palatal or lingual abnormalities; assessment of occlusion and eruption patterns; and evaluation for missing, delayed, or malformed teeth. All findings were recorded in a structured case report form and photographed, with consent, for expert review.

### Statistical analysis

All statistical analyses were performed using a significance threshold of α=0.05. Categorical variables, including prenatal-visit categories, newborn VDRL titers, need for neonatal treatment, maternal educational level, dental pain frequency, and dental-clinic attendance, were compared among the untreated, treated, and control groups using the chi-square test of independence. When expected cell counts were <5, Fisher's exact test was used. Continuous variables, including maternal age, were assessed for normality using the Shapiro-Wilk test. Because maternal age was normally distributed across groups, mean values were compared using one-way analysis of variance, followed by Tukey's post hoc test when applicable.

## RESULTS

In Phase 1, maternal age did not differ substantially across groups (Table 1). Most mothers in all groups were 19-35 years old. The untreated group had the highest mean age (26.4±6.2 years), followed by the control group (24.6±5.2 years) and the treated group (23.6±4.6 years). Although the mean values differed numerically, no statistically significant between-group difference was observed (p>0.05).

**Table 1:** Maternal age and educational level in Phase 1

		Untreated (n=12)	Treated (n=17)	Control (n=10)
Age (years)				
	<18 years	3	2	1
	19–35 years	9	15	9
Mean (SD)		26.4 (6.2)	23.6 (4.6)	24.6 (5.2)
Educational level				
	Incomplete elementary school	3	9	2
	Completed elementary school	1	0	0
	Incomplete high school	3	1	3
	Completed high school	4	5	4
	Completed higher education	1	2	1

Maternal educational level did not differ significantly across groups (χ²=7.54; p=0.480; Table 1). However, educational attainment in the untreated group was broadly distributed, with many mothers reporting incomplete elementary or high-school education. The treated group had a higher proportion of mothers who had completed high school than the untreated group. The control group had the highest proportion of mothers with completed higher education, whereas this category was less frequent in the syphilis-exposed groups.

In Phase 1, the number of prenatal visits differed across groups (Table 3). Dental-clinic attendance also differed significantly across groups (χ²=6.93; p=0.031; Table 2), with the lowest attendance observed in the treated group (2/17). Attendance was slightly higher in the untreated group (4/12). The highest attendance was observed in the control group, in which 6/10 mothers attended a dental clinic. Overall, mothers in the syphilis-exposed groups appeared less likely to access dental care than those in the control group. Reports of dental pain varied numerically across groups: 1 mother in the untreated group reported dental pain. In the treated group, 5 mothers reported dental pain. In the control group, no mothers reported dental pain. Clinical variables were compared among the untreated (n=12), treated (n=17), and control (n=10) groups using the chi-square test. By contrast, the presence of dental pain did not differ significantly among groups (χ²=4.85; p=0.089), although reported pain was numerically more frequent in the treated group than in the untreated and control groups. These findings indicate that dental-clinic attendance, but not dental pain, differed significantly among groups.

**Table 2:** Maternal dental-clinic attendance and dental pain in Phase 1

		Untreated (n=12)	Treated (n=17)	Control (n=10)
Dental-clinic attendance				
	Yes	4	2	6
	No	8	15	4
Dental pain				
	Yes	1	5	0
	No	11	12	10

**Table 3:** Prenatal visits and syphilis diagnosis during pregnancy in Phase 1

		Untreated (n=12)	Treated (n=17)	Control (n=10)
Prenatal visits				
	None	5	1	2
	<5 visits	0	3	6
	6–10 visits	5	11	2
	>11 visits	2	2	
Diagnosis during pregnancy				
	Yes	7	16	Not applicable
	No	5	1	Not applicable

Table 3 shows prenatal-visit distribution across groups. The number of prenatal visits differed significantly among groups (χ²=12.84; p=0.012), with untreated mothers less likely to attend adequate prenatal care than treated and control mothers. Diagnosis of syphilis during pregnancy differed significantly between the untreated and treated groups (χ²=6.32; p=0.012), with treated mothers more frequently diagnosed during pregnancy than untreated mothers. These findings indicate differences in prenatal care access and diagnostic coverage that may have contributed to different congenital syphilis outcomes across groups.

Newborn venereal disease research laboratory (VDRL) titers (Table 4) differed significantly among groups (χ²=22.84; p<0.0001). Infants in the untreated group had the widest titer range, including values of 1:128 and 1:512. The treated group showed predominantly low titers (mainly 1:2 and 1:4), with five non-reactive newborns, whereas all infants in the control group were non-reactive. The between-group distribution of reactive versus non-reactive VDRL results was also significant (χ²=31.27; p<0.001). The proportion of newborns requiring treatment did not differ significantly between the untreated and treated groups (χ²=0.13; p=0.72). Polymerase chain reaction (PCR) testing identified *Treponema pallidum* DNA in four newborns: three in the untreated group, with VDRL titers of 1:32 and 1:512, and one in the treated group, with a titer of 1:4. Together, these findings show lower VDRL titers and fewer reactive results in newborns of treated mothers, although treatment did not eliminate the need for neonatal intervention or the detection of treponemal DNA.

**Table 4:** Newborn syphilis diagnosis and treatment in Phase 1

		Untreated (n=12)	Treated (n=17)	Control (n=10)
Newborn VDRL titer				
	1:2	3	4	0
	1:4	2	6	0
	1:8	2	0	0
	1:16	2	1	0
	1:32	0	1	0
	1:128	1	0	0
	1:512	2	0	0
	Non-reactive	0	5	10
Newborn treatment required				
	Yes	9	13	Not applicable
	No	3	4	Not applicable

In Phase 2, five children aged 5-7 years were evaluated. The mean maternal age at the time of childbirth was 24.4 years. All mothers reported receiving at least three doses of benzathine penicillin, and all children received postnatal treatment. The children's oral hygiene status was classified as fair to poor, with a mean DMFT of 3.4. All children had dental caries, including cavitated lesions. Among dental alterations described in association with congenital syphilis, enamel hypoplasia was identified in four of the five children. Two children exhibited dental alterations typically associated with congenital syphilis, including Hutchinson's incisors and mulberry molars. These findings are illustrated as mulberry molars in figure 1 and Hutchinson's teeth in figure 2.

**Figure 1:**
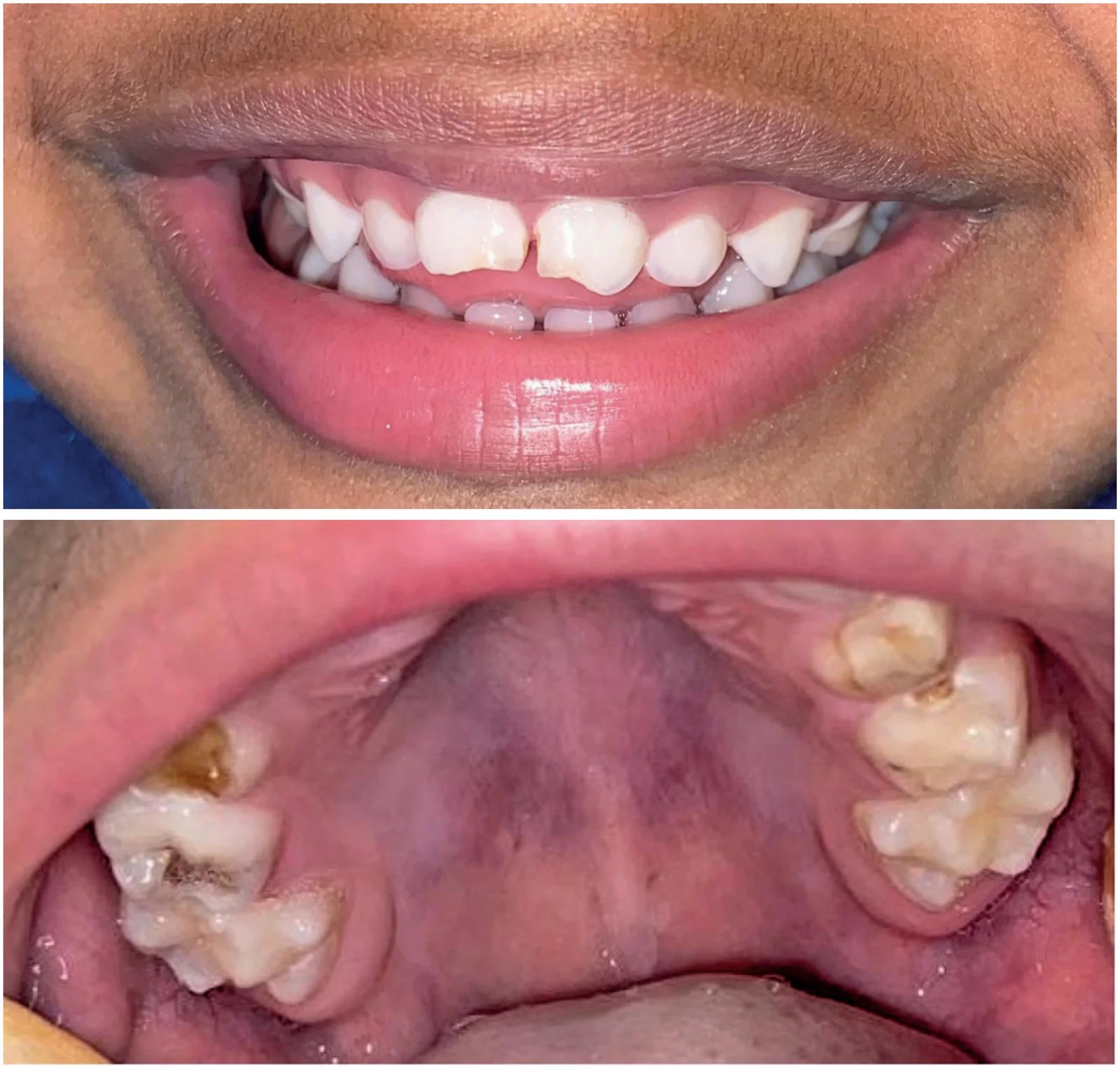
Mulberry molars observed during the Phase 2 oral examination

**Figure 2:**
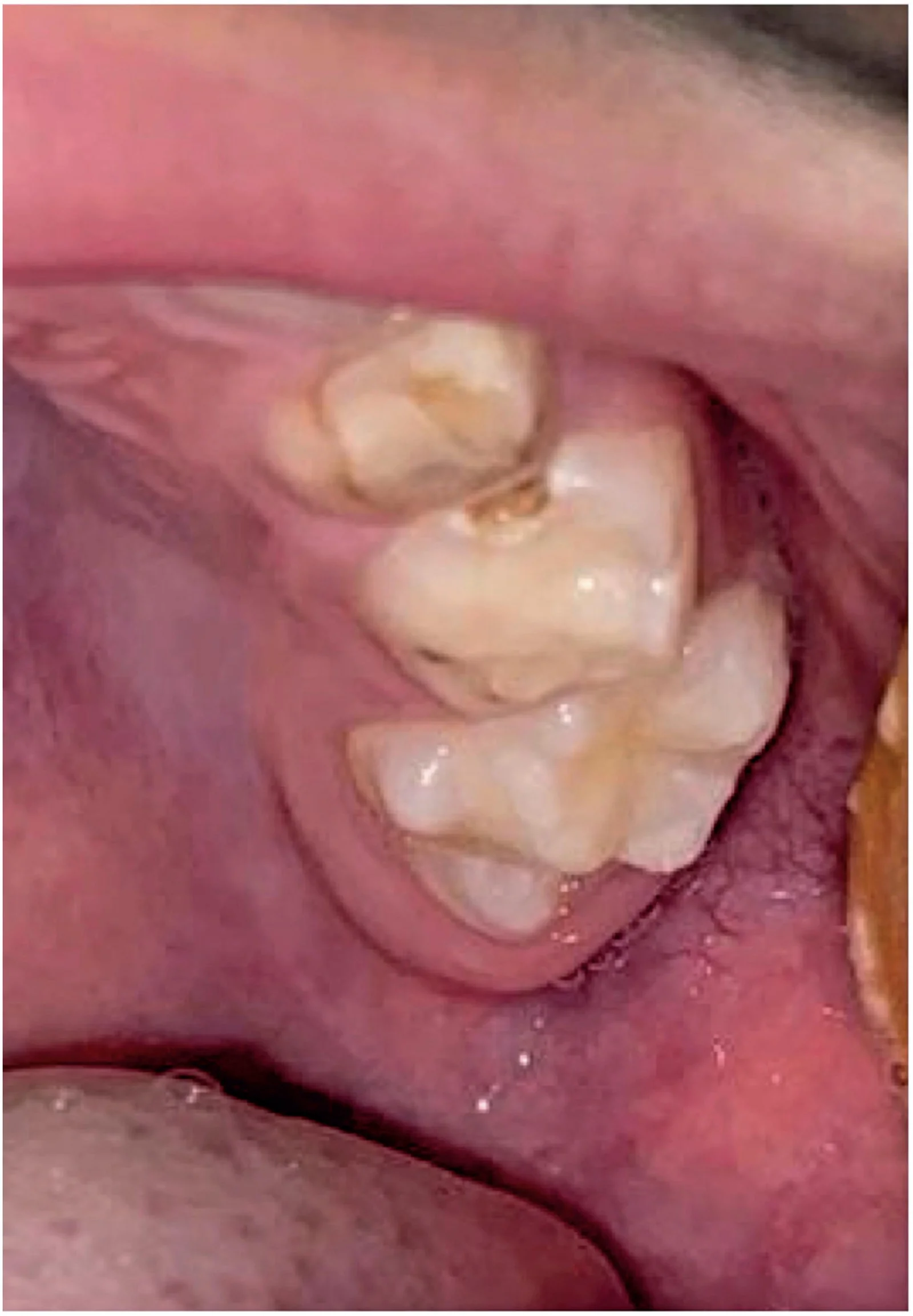
Hutchinson's teeth observed during the Phase 2 oral examination

## DISCUSSION

This study provides a multifaceted analysis of congenital syphilis by integrating social, maternal, neonatal, and oral-health outcomes, thereby illustrating persistent challenges in the prevention and follow-up of this infection. Our findings are consistent with reports describing the resurgence of syphilis and congenital transmission despite the longstanding availability of diagnostic tools and penicillin treatment.^(1,2)^ Consistent with contemporary epidemiological reports, our data suggest that CS reflects systemic inequities, particularly gaps in antenatal-care access, maternal screening, and timely treatment, which are recognized determinants of vertical transmission.^(10-12)^

In Phase 1, differences were observed among the untreated, treated, and control groups in educational level and prenatal-care attendance. Mothers in the untreated group had the lowest number of prenatal visits, with nearly half reporting no prenatal care. This pattern is consistent with international evidence linking inadequate antenatal follow-up to CS.^(8,9)^ The lower educational attainment observed in the untreated group is also consistent with reported associations between social vulnerability and increased risk of maternal syphilis.^(3)^

The difference in prenatal diagnosis rates between groups—58% in the untreated group versus 94% in the treated group—also reflects the role of screening gaps in ongoing fetal exposure.^(4)^ This gap is clinically relevant because congenital infection can often be prevented through timely maternal diagnosis and appropriate penicillin therapy.^(6,8)^

Neonatal venereal disease research laboratory titers in Phase 1 showed a gradient according to maternal treatment status. High titers were observed only among infants in the untreated group, whereas the treated group showed predominantly low titers or non-reactive serology. These findings are consistent with evidence that maternal treatment reduces fetal treponemal burden and disease severity.^(4,7)^ The molecular findings also suggest an association between higher serological reactivity and detectable treponemal DNA, supporting the possibility that untreated or inadequately treated maternal infection may be associated with residual treponemal genetic material in neonatal oral samples.

The need for neonatal treatment in both exposed groups suggests that maternal therapy reduces but may not eliminate the risk of fetal infection, particularly when treatment is delayed or incomplete.^(13,14)^ This consideration is relevant when interpreting the Phase 2 findings on dental development.

A notable Phase 2 finding was the presence of dental stigmata among children aged 5-7 years who had reportedly received both maternal and postnatal therapy. Four children had enamel hypoplasia, and two exhibited classic dental anomalies associated with late congenital syphilis: Hutchinson's teeth and mulberry molars. These defects have been described in relation to CS and remain important clinical markers of historical treponemal infection.^(16,17,19,20)^

These malformations are related to the timing of fetal tooth development. When *T. pallidum* infection occurs during critical developmental windows, ameloblast function may be disrupted, resulting in enamel hypoplasia and aberrant cusp formation, even if the infection is subsequently treated.^(23)^ These defects may therefore represent irreversible developmental sequelae of past treponemal injury.

The persistence of these anomalies is consistent with the principle that penicillin can prevent stillbirth and early systemic morbidity but cannot reverse developmental damage already established in utero.^(15)^ Historical and modern studies indicate that dental stigmata are among the recognized late manifestations of CS.^(21,24)^

The presence of mulberry molars and Hutchinson's incisors in treated children supports the role of dental professionals in the syphilis detection and follow-up continuum. Oral lesions are recognized clinical signs in both acquired and congenital syphilis,^(22,27)^ and dentists may help identify evidence of past congenital infection.

Because late manifestations may appear years after neonatal serology has normalized, dental evaluation may serve as an adjunct to traditional screening and follow-up. Paleopathological and clinical literature indicates that skeletal and dental findings may retain evidence of treponemal infection after other signs have resolved.^(17,18)^

In addition to syphilis-associated findings, all children had carious lesions and cavitated teeth, with a mean decayed, missing, and filled teeth (DMFT) index >3, suggesting broader oral-health vulnerability. Mothers in the syphilis-exposed groups had lower dental-attendance rates, consistent with evidence linking socioeconomic disadvantage and poor childhood oral health.^(26)^

Although polymerase chain reaction (PCR) positivity was identified in oral samples, its significance regarding bacterial viability remains uncertain. Prior research suggests that mucosal and oral niches, including dental pulp, gingival sulci, and salivary glands, may harbor treponemal DNA after treatment.^(28-30)^ Whether molecular positivity reflects residual non-viable DNA fragments or early-life colonization remains unclear. Nonetheless, the observed association between higher VDRL titers and PCR positivity supports further evaluation of molecular tools as complementary methods for monitoring children exposed to CS.

## CONCLUSION

Congenital syphilis remains an important public-health problem associated with gaps in maternal screening, prenatal care, and socioeconomic support. In this study, untreated mothers had fewer prenatal visits, and neonates born to untreated mothers showed higher venereal disease research laboratory titers, highlighting the importance of antenatal diagnosis and timely treatment. The detection of *Treponema pallidum* DNA in oral samples, including in participants exposed to maternal and neonatal treatment, supports the potential value of molecular methods such as PCR for identifying residual treponemal DNA. These findings support the importance of strengthening prenatal care, universal maternal screening, integrated pediatric-dental follow-up, and further evaluation of PCR as a complementary tool for monitoring possible long-term oral sequelae of congenital syphilis.

## Data Availability

After publication, data will be available from the authors upon request—this condition is justified in the manuscript. Due to ethical restrictions involving patient data, the dataset is not publicly available but may be obtained from the authors upon reasonable request.
